# Co-expression network analysis and genetic algorithms for gene prioritization in preeclampsia

**DOI:** 10.1186/1755-8794-6-51

**Published:** 2013-11-12

**Authors:** Eduardo Tejera, João Bernardes, Irene Rebelo

**Affiliations:** 1 Institute for Molecular and Cell Biology (IBMC), University of Porto, Porto, Portugal; 2 Center for Research in Health Technologies and Information Systems (CINTESIS), Faculty of Medicine, University of Porto, Porto, Portugal; 3 Department of Obstetrics and Gynecology, São João Hospital of Porto; INEB — Institute of Biomedical Engineering, Porto, Portugal; 4 Laboratory of Biochemistry, Department of Biological Sciences, Faculty of Pharmacy, University of Porto, Porto, Portugal; 5 Institute for Molecular and Cell Biology (IBMC), University of Porto, Portugal, Porto, Portugal

## Abstract

**Background:**

In this study, we explored the gene prioritization in preeclampsia, combining co-expression network analysis and genetic algorithms optimization approaches. We analysed five public projects obtaining 1,146 significant genes after cross-platform and processing of 81 and 149 microarrays in preeclamptic and normal conditions, respectively.

**Methods:**

After co-expression network construction, modular and node analysis were performed using several approaches. Moreover, genetic algorithms were also applied in combination with the nearest neighbour and discriminant analysis classification methods.

**Results:**

Significant differences were found in the genes connectivity distribution, both in normal and preeclampsia conditions pointing to the need and importance of examining connectivity alongside expression for prioritization. We discuss the global as well as intra-modular connectivity for hubs detection and also the utility of genetic algorithms in combination with the network information. *FLT1*, *LEP*, *INHA* and *ENG* genes were identified according to the literature, however, we also found other genes as *FLNB*, *INHBA*, *NDRG1* and *LYN* highly significant but underexplored during normal pregnancy or preeclampsia.

**Conclusions:**

Weighted genes co-expression network analysis reveals a similar distribution along the modules detected both in normal and preeclampsia conditions. However, major differences were obtained by analysing the nodes connectivity. All models obtained by genetic algorithm procedures were consistent with a correct classification, higher than 90%, restricting to 30 variables in both classification methods applied.

Combining the two methods we identified well known genes related to preeclampsia, but also lead us to propose new candidates poorly explored or completely unknown in the pathogenesis of preeclampsia, which may have to be validated experimentally.

## Background

Preeclampsia remains a leading cause of maternal/fetal mortality and morbidity associated with gestational hypertension and proteinuria. The underlying mechanism and preventive treatment [1,2] remain unknown and therefore, it is still known as the “disease of theories” [3]. Due to possible multifactorial causes involved [1,2,4], an increase in “omics” experimental approaches is noted, generating a large amount of information, not always integrated or analysed by recent methodologies.

Some bioinformatics analysis were performed on specific microarray assays [5-7], and our group has recently carried out an extensive review of related data, processing multiple microarrays combined with text mining tools that led to the identification of several specific genes [8].

In this work, we present a different strategy focused on gene prioritization by co-expression network analysis and genetic algorithms optimization. We also increase the number of microarrays processed.

## Methods

### Microarray processing

Experimental microarray data comparing normal (N) and preeclamptic pregnancies (PRE) was obtained analysing the Gene Expression Omnibus (GEO) and ArrayExpress databases [9,10]. Only the studies comprising more than 10 subjects (by groups) were included (Table 1).

**Table 1:** General microarrays information

**Code**	**Database**	**Sample**	**Method**	**Tissue**	**Ref.**
E-TABM-682	Array express	13(PRE), 58(N)	Illumina	Placenta	[11]
E-MEXP-1050	Array express	16(PRE), 17(N)	Affymetrix	Placenta	[12]
GSE25906	GEO	23(PRE), 37(N)	Illumina	Placenta	[13]
GSE14722^2^	GEO	12(PRE), 11(N)	Affymetrix	Placenta	[14]
GSE10588	GEO	17(PRE), 26(N)	ABI Human	Placenta	[7]

Notes: All samples were collected for biopsy of placenta during childbirth. 2) In the GEO appear two platforms (GPL96, GPL97) but only GPL96 was used because a greater number of probes are shared with other platforms.

Table 1 shows the GEO and Array Express data sources, references, and additional information used in the study. Each microarray was processed as follows: for Affymetrix platforms, the raw data was *mas5* preprocessed and log2 transformed using *affy* package [15], in Bioconductor [16]; for Ilumina platforms, batch correction, normalization and log2 transformation were performed using the *lumi* package [17], also in Bioconductor; finally, ABI Human platform was used as provided. The authors (GSE 10588, ABI platform) indicated that the arrays were quantile normalized and background correction was performed using ABI 1700 software, however, we extracted and processed the public data using *GEOquery* package [18] in Bioconductor.

In cross-platform microarray analysis the first step, after individual microarrays analysis is to combine the different probes. For this task usually a common identifier is used (i.e. entrez gene, unigene code) in order to obtain the common space across all platforms [19-21]. We mapped the arrays probes for the respective entrez gene ID through manual observation and also using the updated manufacturers annotation information (using R-packages: *lumiHumanIDMapping* and *hgu133b.db*[22,23]) for all platforms. Only genes common to all platforms (6816 genes) were used in the subsequent analysis. Genes with more than one probe were combined by averaging the intensity values using *collapseRows* and *intersect* the functions available in the *WGCNA* package [24,25]. The second normalization was performed in order to re-scale the intensity and also remove cross-platform batch effects using *Combat* function in *SVA* package [26]. The identification of genes with statistically different expression between N and PRE groups was performed using *lmFit* from *Limma* R-Package [27] and only genes with p ≤ 0.05 (n = 1146 genes) were considered for co-expression networks construction.

### Co-expression network construction and analysis

Genes differentiated (n = 1146 genes) between N and PRE groups were used for weighted genes co-expression (CoE) network construction in each group using *WGCNA* package [24]. In the weighted genes co-expression network the nodes represent genes and the edges represent the connection strength which corresponds essentially to a weighted adjacency matrix (A) with elements α_*i*,*j*_ = |*cor*(*x*_*i*_, *x*_*j*_)|^*β*^ where x_i_ and x_j_ are the expression profiles of genes *i* and *j*. This method considers a continuous ([0,1] interval) instead of discrete adjacency matrix which proved to be highly robust with respect to the β parameter variation.

**Figure 1:**
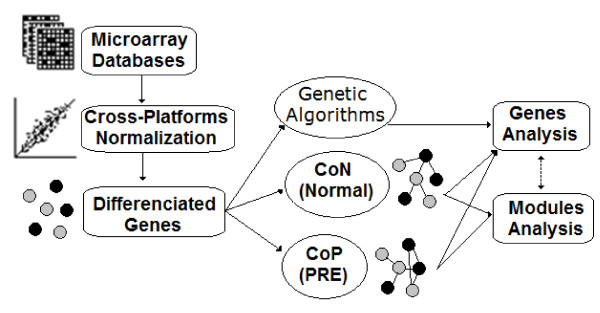
**Workflow overview representing the different procedures explored in the present study.** CoN and CoP: Normal and Preeclampsia co-expression matrixes, respectively.

In this study, we selected β = 6, following the scale-free topology criterion proposed by Zhang and Horvath using the *pickSoftThreshold* function in WGCNA (data not shown) [28]. Once defined the adjacency matrix for each group (normal and PRE), the co-expression matrix (CoN and CoP, respectively) and the topological overlap matrix (TOM) were obtained (Figure 1). The topological overlap matrix (TOM) is the central starting point for network modules detection and analysis and each element (ω_ij_) represents a measure of similarity between two nodes in the same network.

Further analyses were divided into main branch (Figure 1): a) modular (inter and intra-modules) analysis and b) genes (nodes) analysis.

### Modules analysis

The modules were detected using the Dynamic Tree Cut algorithm [29] with *cutreeDynamic* function in WGCNA package and defining the deep split = 3 and cutting height corresponding to the 99th percentile and the maximum of the joining heights on the dendrograms. In each module, the node connectivity and the node intra-modular connectivity were calculated. This means, the node connectivity is basically defined as the sum of the weights of all edges connected to it (*k*_*i*_ = ∑ _*j*_α_*i*,*j*_), however, when a module is defined each gene is now linked with a specific subgroup of neighbours and therefore connectivity will change as a consequence of reduction of the number of neighbours, therefore, in a network with m = 1,2,…,M modules and where each of these modules has *N*_*m*_ nodes, the intra-modular connectivity ($k_{i}^{m}$) is defined as $k_{i}^{m}=\sum_{j}^{N_{m}}a_{i,j}$, this means, the sum of the weights of all edges connected to node *i* in module *m*.

We also compared the modules obtained for each network (CoN and CoP), using the Fisher’s exact test, where we basically analysed the number of common genes between modules. Therefore, the modules (between the two conditions), with an increased number of shared genes tend to be more unspecific. Combined analysis of intra-modular connectivity and modules comparison could provide a better identification and description of each gene in the network modules, and therefore the identification of potential network hubs.

#### Nodes analysis

Even when the module analysis above could provide a deep inside in the network hubs in identifying PRE specific related genes, we are interested in a direct comparison between nodes (genes) in both networks. The weighted adjacency matrix represents a complete graph because each element of the network is connected (even with very low strength), moreover, both the network have the same number of nodes and edges, so if connectivity environment of a gene *i* is similar in both network then the genes are also similar in both physiological states. Consequently, we define a distance measure of gene *i* between two networks (CoN and CoP) as follows:

$$\mathrm{KDis}t_{i}=\frac{1}{k_{i}^{\mathrm{CoP}}+k_{i}^{\mathrm{CoN}}}\sqrt{\sum_{j=1}^{N}{\left(\alpha_{i,j}^{\mathrm{CoP}}-\alpha_{i,j}^{\mathrm{CoN}}\right)}^{2}}$$

The highest values of *KDist* correspond to genes with very different connectivity environments and therefore more likely to be a significant gene in the PRE condition. Similar procedures were implemented by other authors, using the node degree obtained in binary adjacency matrix and counting the number of common edges [30,31]. To select a group with increased distance values, we need to identify a distance cut-off. The cut-off distance was selected for comparison with 1000 randomized network as follows: for each network (CoN and CoP) 1000 network were obtained by random permutation of the original edges strengths (a_i,j_). Among all networks (randomized N and PRE) the *KDist* is computed followed by counting the number of nodes with distances higher than a predefined percent (cut-off value) of the maximum distance value. The selected numbers of genes for the different cut-off values were compared using t-test (similar strategy was followed in [30,31]).

### Gene ontology and metabolic pathway enrichment analysis

The gene ontology and pathways enrichment analysis were performed using DAVID bioinformatics resource v6.7 [32], exploiting the well know databases: gene Ontology and KEGG databases. Complete enrichment analyses of each of the network modules will considerable increase the length of the presented work and therefore we will present only the results obtained for specific and relevant modules.

### Genetic algorithm optimization in genes selection

These procedures can also be seen as a node centred analysis, but considering exclusively expression values and not a network structure. The general idea is to identify genes (combination of them) that maximize the differentiation between N and PRE groups. In this context, we can apply a combination of genetic algorithm (GA) optimization with two widely used classification methods: Nearest Neighbour (GANN) and Discriminant Analysis (GADA). We used the Euclidian as the distance metric in the nearest neighbour algorithm and the linear discriminant function in the discriminant analysis. It is important to realize that other metrics can be used in both the nearest neighbour and discriminant analysis and probably will lead to different results, however, find the “best” strategy is not an objective of the present study and probably will depend on particular classification/data problem.

The GA was run 25 times with different initial populations and each of the final models was used in further analysis. The GA initial parameters were: 1,000 generations, the initial population of 100 chromosomes and a cross-over and mutation probability of 0.7 and 0.3, respectively. The maximum number of selected genes was restricted to 30. The criterion for model selection was the leave-one-out (LOO) cross-validation procedure and therefore, for each algorithm, we have a set of 25 models and the error estimated by the LOO, respectively.

The 25 models obtained in each algorithm procedure (a total of 2x25 models and a maximum possibility of 25x30 different genes, by procedure) do not comprise the same genes but a space of them. However, some genes are frequently present across the models and therefore may be of specific interest in further considerations. We also cross-analysed the genes space obtained by the different GA procedures with the respective gene location in the network modules and also the relationship with *KDist* leading to integrated information and facilitates the interpretation.

## Results

**Figure 2:**
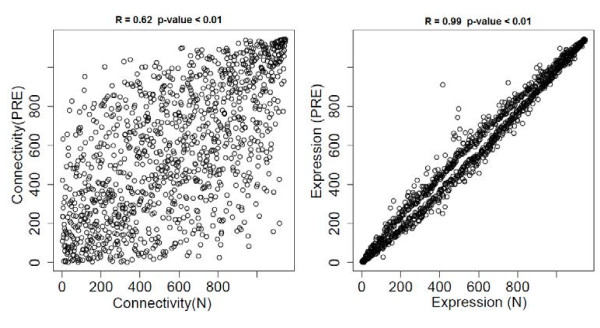
Left: Mean ranked expression comparison; Right: Mean ranked connectivity comparison between Normal and PRE group.

The correlation between the mean ranked expression, as well as the mean ranked connectivity between N and PRE groups shows a higher correlation for the expression instead of connectivity, even when both are statistically significant (Figure 2).

The average nodes degree in each network were (mean ± standard error): 1.57 ± 0.05 (N) and 32.7 ± 0.19 (PRE), indicating that the correlation between the gene expression in PRE is increased. This means that the interconnectivity between genes is higher on PRE over N.

**Figure 3:**
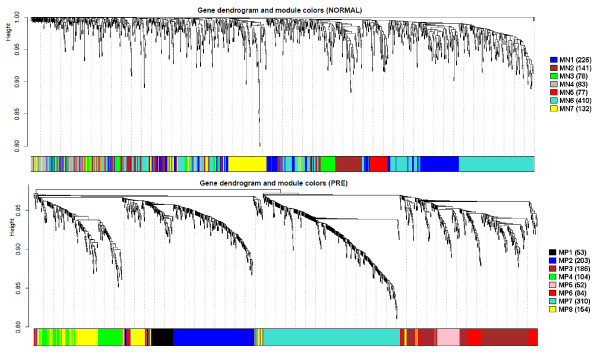
Dendrograms and module representations obtained in normal (Top) and preeclamptic (Botton) co-expression networks.

Using the Dynamic Tree Cut method, 8 and 7 modules were identified in CoP and CoN, respectively (Figure 3) marked as follows: CoP) MP1 (53 genes), MP2 (203 genes), MP3 (186 genes), MP4 (104 genes), MP5 (52 genes), MP6 (84 genes), MP7 (310 genes) and MP8 (154 genes); CoN) MN1 (225 genes), MN2 (141 genes), MN3 (78 genes) MN4 (83 genes), MN5 (77 genes), MN6 (410 genes) and MN7 (132 genes). The MN4 (grey) correspond to those genes that are not grouped in any particular community.

**Figure 4:**
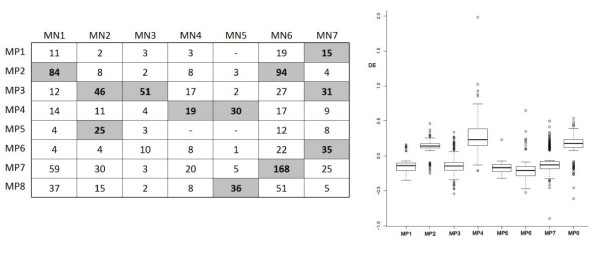
Left: Fisher’s exact test in modules superposition where coloured cells represents p-value <0.05; Right: CoP modules differential expression (DE).

The comparative enrichment module analysis reveals that all modules have a certain overlap between N and PRE group (Figure 4 Left) to some degree, suggesting that the genes are grouped in a similar fashion between the two conditions. Furthermore, we note that the MP4 comprise genes with large differences in the expression values (mainly up-regulated), suggesting that this module could include genes of interest (at least in terms of expression). In fact, the gene ontologies and pathways enrichment analysis indicates that biological processes like (p-value < 0.01): ovulation cycle, sexual differentiation, regulation of hormone levels and the erythrocyte differentiation are significantly enriched in the MP4 module, as well as metabolic pathway related with the GnRH signalling pathway that is closely related with the cytokine-cytokine receptors interactions. Hormonal modifications and cytokine signalling processes are highly relevant in the PRE (see Discussion).

Similar modules relevance can be considered for MP6 and MP8 because they comprise (especially MP6) mainly down-regulated genes. Genes in MP6 module enrich biological processes as (p-value < 0.01): vascular development, blood vessel development, vasoconstriction, cell adhesion, regulation of endothelial cell proliferation as well as metabolic pathways (p-value < 0.01) like ECM-receptor interactions and focal adhesion. All these processes are relevant and well known in PRE.

Besides considerations of expression values we must also consider the way in which genes are connected, actually, even when the modules overlap is significant, a different picture emerges by restricting the overlap to genes with increased intra-modular connectivity $\left(k_{i}^{m}>\langle k_{i}^{m}\rangle +\mathrm{std}\left(k_{i}^{m}\right)\right)$. Table 2 shows the top 10 genes with increased intra-modular connectivity (some modules do not have 10 tops and hence a reduced number is displayed), where we may notice that a few genes overlap between modules (only 28 genes of 145 selected higher). These results suggest that a large amount of common genes are not highly connected or, more importantly, they are highly related, but only in one of the networks.

**Table 2:** Modular analysis

PRE	MP1	EEF2,GSTK1,ATP5I,MRPL12,NME4,EFEMP2,PCOLCE,RASL12,ADD1
	MP2	AP2M1,AP2B1,TTC1,ACTR1A,MED12,TAF10,VPS72,CSNK2B,ATOX1,UBTF
	MP3	CNIH,B3GNT2,RAN,GTF2E1,RB1,ME2,CRTAP,PNMA1,IDH1,RPL15
	MP4	FSTL3,FLT1,TPBG,NDRG1,INHA,HEXB,ENG,INHBA,FLNB,SPAG4
	MP5	TSNAX,SYPL1,PSMA1,PRDX3,DCK,PUM1,CTNNB1,ZNF217,PSMC6,CSNK1A1
	MP6	TAGLN,MYH11,COL1A2,WFDC1,COL1A1,ACTA2,ACTG2,DPYSL3,CDH5,PDLIM3
	MP7	DLD,SFRS10,UQCRC2,PRKRA,CCNG1,DNAJB4,STRAP,SEPT2,RBBP7,SEPP1
	MP8	EZR,KRT19,CYP11A1,TECR,DDR1,PPP1R13L,SLC35A2,ILVBL,ARID3A,EPHB3
NORMAL	MN1	NAP1L1,MGAT1,CMPK1,HOXA10,CTSD,RBBP7,UPF1,ATP6V0B,NR1H2,NPTN
	MN2	ZFAND6,JAK1,TSNAX,KLF10,RRAS2,MAP4K3,PSMA1,FNTA,SYPL1,MAEA
	MN3	CNIH,RB1,PMP22,IDH1,CAV2,CRTAP,IL33,HPRT1,RPL15,PDGFRA
	MN4	MS4A6A,CYBB,TLR7,FOS,DUSP1,FOSB
	MN5	CYP11A1,EZR,DDR1,LAD1,TECR,KRT19,ELF3,CLDN7,ILVBL,SPINT2
	MN6	DLD,UQCRC2,MORF4L1,SEPT2,SNAP23,CCNG1,ZNF12,TWF1,NFE2L2,SEP15
	MN7	TAGLN,MYH11,COL1A2,ACTG2,DPYSL3,WFDC1,ACTA2,PDLIM3,TNC,RASL12

Selected genes with the highest intra-modular connectivity.

MP7 module showed 168 significant overlapped genes with MN6. However, after the connectivity restriction few remained, even when both MP7 and MP6 are modules with higher intra-modular connectivity (Table 3). This suggests that MP7 could be a potential module for hubs identification. In addition, some of the selected genes, for example in MP4, are well known in relation with PRE (i.e. *INHA*, *FLT*, *ENG* see Discussion) and that are not shared with the CoN modules.

**Table 3:** Modules statistics

**CoP modules**	**<** ***k*** **>**	$\langle k_{i}^{m}\rangle$	**CoN modules**	**<** ***k*** **>**	$\langle k_{i}^{m}\rangle$
MP1	31.68	4.24	MN1	1.75	0.83
MP2	32.41	13.31	MN2	1.25	0.58
MP3	33.61	11.61	MN3	0.95	0.29
MP4	30.92	8.41	MN4	0.14	0.02
MP5	35.15	4.29	MN5	1.08	0.39
MP6	31.28	5.20	MN6	2.29	1.62
MP7	34.04	20.41	MN7	0.95	0.49
MP8	31.06	7.96			

The variation of $\langle k_{i}^{m}\rangle$ tend to be higher than <k> and not necessarily expresses the same behaviour for both, i.e. the MP5 modules showed the highest <k> values but almost the minimal $\langle k_{i}^{m}\rangle$ones. Modules with increased intra-modular connectivity suggest genes involved in similar process, however, in terms of significance, the modules (or differentiability) on the intra-modular connectivity cannot be considered without global connectivity. In terms of expression differentiability, MP4 module can be considered relevant because of the higher expression differences (Figure 4 right) but considering the connectivity aspects we should include MP7 by the higher intra-modular connectivity. It is precisely this dichotomy between expression and connectivity that leads us to consider further based gene analysis.

The enrichment analysis for the MP5 and MP7 modules indicates less specific processes that can agree with the highest <k> in both modules. The gene ontology database has a hierarchical structure where the specificity of the biological processes increases at each ramification level. Modules such as MP4 and MP6 previously discussed are significantly enriched in biological processes, even at level 4 or 5 leading us to specific (and therefore more relevant) processes, while a different pattern emerges from MP7 and MP5.

Most of the statistically significant biological processes enriched in MP5 module can’t go further than level 2 (i.e., cell cycle process and organelle organization), and therefore is expected a high <k> but lower $\langle k_{i}^{m}\rangle$ as shown in Table 3. Moreover, the genes in MP7 modules enrich a great number of processes in the level 1 and level 2, but also at level 4 and 5 likes (p-value < 0.01): protein folding, DNA metabolic processes and protein transport. These processes related with MP7 module even when specifics are actually part of many central pathways and therefore we should expect a high <k> and $\langle k_{i}^{m}\rangle$ as shown in Table 3.

**Figure 5:**
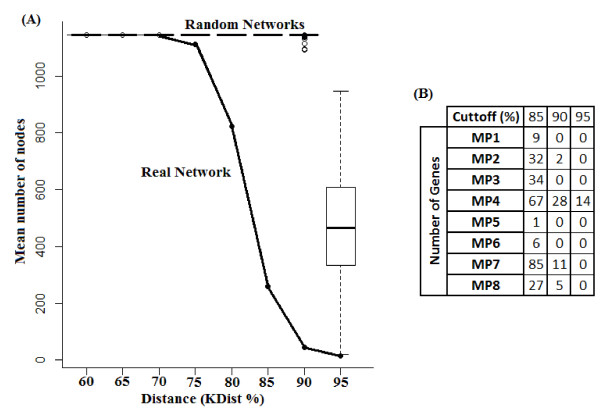
**Distance cutoff analysis (A) and genes distribution across modules (B) for different cutoff values.** The distance cutoff is expressed as the percent of the maximal distance.

Analysing nodes (genes) distance between the two networks (Figure 5), it can be noted that even for relatively low values of distance cut-offs (expressed as percentage of the maximum distance), the number of genes obtained were statistically significant (p-value < 0.01 at 75%) compared to the cut-off of the same randomized networks. For a cut-off distance > 85, 90, and 95% we identified 261, 46 and 14 genes, respectively, distributed in several modules. As can be seen the representative modules according to *KDist* are the MP4 and MP7 considering the biggest differences between the node distances and also in correspondence with the previous modules analysis. In fact, in the 14 genes with maximum *KDist* values- *NDRG1*, *FLT1*, *TPBG*, *FSTL3*, *FLNB*, *INHBA*, *SPAG4*, *INHA*, *HK2*, *HEXB*, *TPI1*, *BCL6*, *LEP*, *QSOX1*- we also found *FLT1*, *FLNB*, *INHA*, *LEP* and *INHBA*, which are some of the nodes with greater intra-modular connectivity.

The analysis of genes based on GA algorithms also indicates a strong participation of MP4, but also including MP1 with GADA (Table 4). Moreover, we found at least one model with more than 97% of correct classification using the GADA procedure while all models revealed a LOO value higher than 90%, with 30 selected genes. The MP1 inclusion as a relevant module is a result of expression exclusive analysis. This means, GA methods consider only the expression values in the model generation and selection of variables and therefore modules like MP7 with a higher intra-modular connectivity, but lower differential expression may be underestimated.

**Table 4:** Genetic algorithm methods results and genes distribution across PRE co-expression network modules

**Methods**	**<LOO>**	**LOO**_**max**_	**MP1**	**MP2**	**MP3**	**MP4**	**MP5**	**MP6**	**MP7**	**MP8**
GANN	0.940	0.965	0.92	1.06	1.07	1.13	1.11	0.91	0.91	0.98
GADA	0.952	0.978	1.54	0.94	1.02	1.31	0.59	1.17	0.90	0.91

Notes: LOO: leave-one-out cross-validation procedure; <LOO> corresponds to the average LOO cross-validation value in the 25 runs; LOO_max_ is the maximum LOO value obtained. The numbers in each of the CoP modules correspond to a ratio of modules that participate in the total 25 models.

Analysing all the genes covered by the 25 models we have: 496 (GANN) and 337 (GADA). The overlap between the two methods led to 163 genes, but only 11 were also found with *KDist* > 90%: *FLT1*, *TPBG*, *FLNB*, *INHBA*, *BCL6*, *QSOX1*, *HILPDA*, *ENG*, *PROCR*, *TTC1* and *SLC6A8*. Most of these genes belong to MP4 modules (n = 10), as expected, due to the influence of this module in expression values, however, being in the top list of *KDist* indicate that these genes also reveal some connectivity contribution. Therefore, these genes are an intermediate point comprising expression and connectivity however, must be plausible to consider the extremes genes in both connectivity (like *INHA*, *NDRG1*, *FSTL3* and/or *RBBP7*) and expression differences (like *MMP1*, *GCLM*, and/or *RARRES2*).

**Figure 6:**
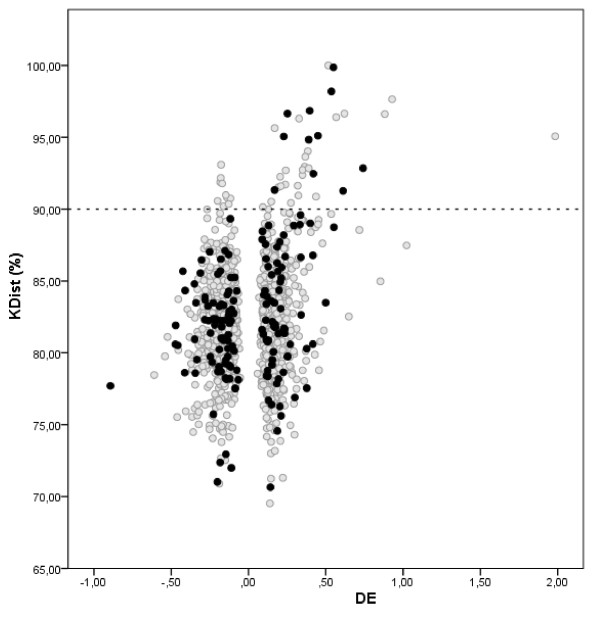
**Variation of KDist with respect to DE for the 1146 considered genes.** The black points correspond to 163 genes obtained by GANN and GADA. There are 46 genes with KDist > 90% located over the horizontal line. The DE represents the mean difference between genes expression in the N and PRE groups.

**Figure 7:**
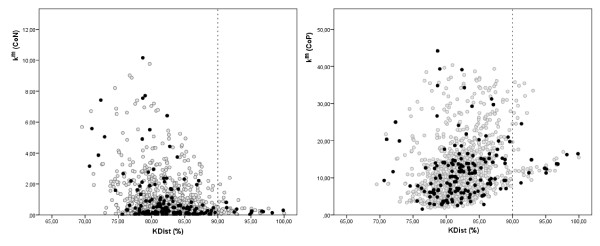
**KDist variation with respect to gene intra-modular connectivity (Left) in normal network (CoN) and (Right) in PRE network (CoP) for the 1146 considered genes.** The black points correspond to 163 genes obtained by GANN and GADA. There are 46 genes with KDist > 90% cuttof marked by the broken line.

To facilitate further discussion we integrated the 163 genes obtained by GANN and GADA superposition in different graphical representations containing information about connectivity, *KDist* and expression (Figures 6 and 7).

Figure 6 shows that the 163 genes obtained by GADA and GANN superposition actually cover a wide range of behaviours expression (down-regulated and up-regulated) and also some of the genes with the highest *KDist* value. Interestingly, the most down-regulated gene (*MMP1*) is covered by GA but not the up-regulated (*LEP*) one. We also can see that the highly up-regulated genes are not necessarily those with the highest topological differentiability. This pattern is even frequent with low regulated genes.

Figure 7 clearly shows that genes with higher intra-modular connectivity are not necessarily those with large topological differences or prioritized by the procedures of genetic algorithms. Even when in CoN there is a clear trend toward for selecting genes with low intra-modular connectivity, in CoP there is no clear trend. Genes with *KDist* > 90% revealed a preference for a minimum intra-modular connectivity in N network but not the maximum in the PRE network.

## Discussion

The lower genes connectivity correlation instead of gene expression intensity between N and PRE groups indicates that genes expression profile is similar in both situations (also partially supported by the similarities between network modules). This means that genes with high or low expression in N are also high or low expressed in PRE. However, the mode by which genes are interconnected or correlated is not so conserved. This also suggests that we have useful information for differentiating normal vs disease by analysing the connectivity network as a complement to an exclusive analysis of genes expression intensity. Furthermore, the increased connectivity degree in the network of PRE group can be a reflection of a highly systemic disorder involving multiple metabolic pathways. This could justify the high probability of multifactorial causes and also the possibility of preeclampsia to progress through more complex clinical conditions [33].

The comparison of network modules between the two conditions revealed that the genes were grouped similarly. All modules revealed some similarities in genes composition between the two conditions. However, there are main differences shown by the way (and strength) by which they are connected. In this regard, the MP7 module is highly similar to MN6 sharing several genes, and many of them also with a high degree of connectivity (i.e. *DLD* and *UQCRC2*) (see Table 2 and Figure 4 left), but it is still more similar to MP6 and MN7 modules sharing a smaller number of genes, but most of them with highest connectivity values (see Table 2 and Figure 4 Left). The average connectivity (total or intra-modular) indicates that MP7 contain genes with strong inter-correlations, however, as we noticed, intra-modular connectivity itself could have misled the module selection in terms of prioritization.

Our results based on *KDist* together with modular analysis indicate that MP4 and MP7 modules comprise the genes with the greatest potential differentiability (Figure 6 Left). In the group of 14 genes with *KDist* > 95% (all of them in the module MP4) we found *FLT1*, *LEP*, *INHA* and *FSTL3*. These genes are well known related to preeclampsia [34-43] and in fact *FLT1* and *INHA* have been used as potential early predictive markers in multivariate models [41-43]. Inhibin B (*INHB*), however, has not been studied, as well as *INHA* during preeclampsia or general pregnancy hypertension and only a previous study indicating significant differences in preeclampsia was found [44]. A similar situation was observed for *BCL6*[45]. Previous publications support an up-regulation of *TPBG*[46] and also *NDRG1*[47]. However, these genes have not been well studied during pregnancy. Other genes as *SPAG4*, *HEXB*, *TPI1* and *QSOX1* are basically unknown in preeclampsia (and even during normal pregnancy). Interestingly, of the 50 genes previously identified in another study and using other network approaches [8], 11 genes were consistent with our *KDist* > 90%, including the little exploited *TPBG*, *NDRG1*, *BCL6*, *LYN* and *FLNB*. Several of these proteins are related to hormone/endocrine pathways (ie, *LEP*, *INHA*, *INHB*), a significant process already highlighted by the enrichment analysis.

The genetic algorithm procedures have led to a high number of genes mainly by using GANN. The nearest neighbour is a non-linear method; therefore, it was not influenced by the variables distribution. However, it is probably by the nearest neighbour approach to select genes that are highly significant (in terms of classification) as a group rather than as individuals [48]. Therefore, given the high co-linearity between genes expression, is quite possible to select a wide range of genes combination capable of achieving a good classification and thus considerably increase the gene space through different models [48]. Even when the best models were obtained with GADA, we think that the main advantage was not in LOO values but in the reduced gene space compared to GANN. On the other hand, the mathematical bases of nearest neighbour classifications are quite different from discriminant analysis. Therefore, the overlapping of both genes spaces, even when functional, may be a drastic approach with risks of rejecting significant genes detected by the methods in their own model space.

Both of these methods GANN and GADA share a large number of genes (163) and some (11) are also present in the *KDist* > 90% group. In this reduced group we found again *FLT1*, *TPBG*, *FLNB*, *INHBA*, *BCL6* and *QSOX1* discussed above, but also other genes such as *ENG* and *PROCR*[34-43,49] which are well documented in preeclampsia. Interestingly some of these genes (i.e. *FLT1*, *FLNB* and *ENG*) are well known related with signalling pathways involved in cytokines interactions and angiogenesis according to the biological processes and pathways enrichment analysis.

These results suggest that the consideration of distance based on the co-expression network connectivity is promising for the identification of significant genes; however, a cutoff of 95% excludes relevant genes. This can be observed not only by the inclusion of genes obtained by GA in the 90% cutoff but also by looking other MP4 genes contained in the range of 90%. In this group we have, for example, the *HTRA1* and *ACVR1* that have been well documented in preeclampsia [50-52].

The genes obtained by GA procedures appear to complement those obtained by *KDist* cutoff > 90% in several ways. Figures 6 and 7 suggest that highly up-regulated genes are better resolved by *KDist* or general topological measurements, contrary to the down-regulated genes. This preference of up-regulated gene to be highly focused was reported by other authors, considering the network interactions [53,54]. However, some of the down-regulated genes, uniquely identified by GA procedures are well documented in studies during PRE as *XBP1* and *MMP1*[55-57] and thus can also be significant to the understanding of the disease and its characterization.

Our results indicate that co-expression network analysis combining both modular and gene centred approaches are capable to identify genes significantly related to preeclampsia. Some of these genes are consistent through genetic algorithms approaches where other down-regulated genes were relevantly prioritized. We were able to corroborate some of the identified genes through manually literature revision in order to validate the hubs identification. However, some other genes remain unexplored or unknown, not only in preeclampsia, but also during pregnancy, leading to the need of further experimental confirmation.

## Conclusions

Genes in weighted co-expression network revealed a similar distribution between detected modules in N and PRE conditions. However, major differences were obtained considering the connectivity of nodules. Genes with more connectivity or intra-modular connectivity were not always detected as network hubs and better results were obtained by comparing the gene and its neighbourhood between the two conditions. In addition, all models obtained by genetic algorithms were consistent with a successful classification higher than 90%, restricting the 30 variables to at least one model greater than 95%.

Gene prioritization from microarray data was improved considering both, gene expression and genes co-expression (connectivity) information. In this sense the co-expression weighted network and genetic algorithms clearly provided consistent and complementary results. Combining the two methods we identified, it is well known preeclampsia related genes like: *FLT1*, *LEP*, *INHA*, *ENG*, *PROCR*, *MMP1*, *XBP1* and *FSTL3*. However, other genes as *FLNB*, *INHBA*, *BCL6*, *TPBG*, *NDRG1*, *LYN* and *QSOX1* were also significant in our analysis, but this has been little explored or is unknown in the current state of the art of preeclampsia pathophysiology. Therefore, these results indicate that more experimental research is warranted to exploit the role of these genes in the development of pregnancy.

## Competing interests

The authors declare that they have no competing interests.

## Authors’ contributions

All authors participated in the study design and coordination. ET performed the network and statistical analysis, as well as drafted the manuscript. JB and IR participated in the discussion of the results as well as the final writing and correction. All authors read and approved the final manuscript.

## Pre-publication history

The pre-publication history for this paper can be accessed here:

http://www.biomedcentral.com/1755-8794/6/51/prepub
